# Bone Mineral Density, C-Terminal Telopeptide of Type I Collagen, and Osteocalcin as Monitoring Parameters of Bone Remodeling in CML Patients Undergoing Imatinib Therapy: A Basic Science and Clinical Review

**DOI:** 10.3390/diseases12110275

**Published:** 2024-11-02

**Authors:** Nurita Indarwulan, Merlyna Savitri, Ami Ashariati, Siprianus Ugroseno Yudho Bintoro, Muhammad Noor Diansyah, Putu Niken Ayu Amrita, Pradana Zaky Romadhon

**Affiliations:** 1 Subspeciality Program in Hematology and Medical Oncology Division, Department of Internal Medicine, Dr. Soetomo General Academic Hospital, Surabaya 60286, Indonesia; nuritaindarwulan1982@gmail.com; 2 Subspeciality Program in Hematology and Medical Oncology Division, Department of Internal Medicine, Faculty of Medicine, Airlangga University, Surabaya 60132, Indonesia; 3 Division of Hematology and Medical Oncology, Department of Internal Medicine, Dr. Soetomo General Academic Hospital, Surabaya 60286, Indonesia; amiashariati@yahoo.com (A.A.); ugrosenoyb2004@yahoo.com (S.U.Y.B.); mnoordiansyah@gmail.com (M.N.D.); nikenamrita@gmail.com (P.N.A.A.); zaky_dr@yahoo.com (P.Z.R.); 4 Division of Hematology and Medical Oncology, Department of Internal Medicine, Faculty of Medicine, Airlangga University, Surabaya 60132, Indonesia

**Keywords:** chronic myeloid leukemia, bone mineral density, bone turnover, tyrosine kinase inhibitor, CTX-1, osteocalcin, Imatinib

## Abstract

Background: Chronic myeloid leukemia (CML) is one of the most commonly found types of myeloproliferative neoplasms, characterized by increased proliferation of granulocytic cells without losing their differentiation ability. Imatinib, a tyrosine kinase inhibitor (TKI), can be effectively used as therapy for CML. However, Imatinib can affect bone turnover thus having clinical implications on the bones of CML patients undergoing long-term Imatinib therapy. However, parameters that can accurately describe the bone condition in CML patients receiving Imatinib still need further study. A combination of imaging techniques such as bone mineral density (BMD) and bone turnover activity markers such as C-terminal telopeptide of type I collagen (CTX-1) and osteocalcin has the potential to be used as monitoring parameters for bone density abnormalities in CML patients receiving Imatinib. Objectives: This article explains the rationale for using BMD, CTX-1, and osteocalcin as monitoring parameters of bone remodeling in CML patients receiving Imatinib. Results: First, the physiological process of bone turnover will be explained. Then, we describe the role of tyrosine kinase in bone metabolism. Next, the impact of Imatinib on BMD, CTX-1, and osteocalcin will be explained. Conclusion: The assessment of bone health of CML patients on Imatinib should include both BMD tests and bone turnover marker assays such as CTX-1 and osteocalcin.

## 1. Introduction

Chronic myelogenous leukemia (CML) is linked to the Philadelphia chromosome, which results from a reciprocal translocation between chromosomes 9 and 22, producing the BCR-ABL chimeric protein with uncontrolled tyrosine kinase activity. This myeloproliferative disorder is characterized by the excessive accumulation of seemingly normal myeloid cells in the bone marrow and peripheral blood. In addition to this myeloid cell buildup, the bone marrow microenvironment in CML also experiences significant changes, such as increased levels of type III collagen (reticulin fibrosis) and enhanced angiogenesis [1].

Protein kinase activity is dysregulated across all cancer types. Imatinib, a tyrosine kinase inhibitor (TKI) that targets ABL, BCR-ABL, PDGFRA, and c-KIT, is widely used in treating chronic myelogenous leukemia (CML) and gastrointestinal stromal tumors (GIST). While Imatinib is typically well-tolerated, its side effects, particularly its impact on bone health, require careful consideration [2]. In a study by Berman et al., it was found that osteocalcin (OCN) levels, an indicator of bone formation by osteoblasts, were reduced in 95% of patients receiving long-term Imatinib therapy, with 37% showing undetectable OCN levels, suggesting diminished bone formation in these individuals. Despite this, only 47% of patients exhibited decreased bone mineral density (BMD), while 21% showed an increase and 32% remained unchanged. The study also identified hypophosphatemia and hyperphosphaturia in 26% of patients, a reduction in serum calcium in 84%, and elevated parathyroid hormone (PTH) levels in 42% of patients. These measurements, including OCN, phosphate, calcium, and PTH levels, were taken during the first two years of Imatinib therapy [3].

Bone density changes in CML patients are typically assessed by measuring bone mineral density (BMD). However, BMD is limited in that it only detects bone disturbances after a significant reduction in bone volume has occurred. In contrast, bone turnover markers (BTMs) can identify abnormalities in bone turnover before changes in BMD become apparent and even independently of BMD variations [4]. A systematic review by Shetty et al. highlighted that BTM measurements are reliable as complementary tools for evaluating microarchitectural changes affecting bone quality, detecting fracture risk, and managing bone density disorders [4]. BTMs are broadly categorized into markers of bone formation—such as osteocalcin (OCN), procollagen type I N-terminal propeptide (PINP), and serum alkaline phosphatase (ALP)—and markers of bone resorption, which include collagen degradation products like collagen type I telopeptides (CTX-1, NTX-1), bone sialoprotein, osteoclastic enzymes, and osteocyte activity markers [5].

Imatinib can influence bone turnover, resulting in clinical effects on the bone health of CML patients undergoing long-term therapy. Detecting changes in bone turnover requires non-invasive assessments, including biochemical markers like CTX-1 and osteocalcin, as well as radiological evaluations of bone mineral density (BMD). This article aims to elucidate the rationale for utilizing BMD, CTX-1, and osteocalcin as monitoring parameters for bone remodeling in CML patients treated with Imatinib. It will first outline the physiological processes of bone turnover, followed by a discussion on the role of tyrosine kinase in bone metabolism and its effect on serum and phosphate levels in CML patients. Subsequently, the article will explore the impact of Imatinib on BMD, CTX-1, and osteocalcin.

## 2. Physiology of Bone Turnover

Bone is a metabolically active structure that undergoes continuous remodeling throughout life. After reaching peak bone mass, bones are consistently remodeled through bone resorption followed by bone formation within basic multicellular units, known as “bone remodeling units.” Under optimal physiological conditions, bone resorption takes approximately ten days, while bone formation requires around three months. Each year, up to 20% of the entire skeleton is replaced through this remodeling process [4,6,7].

The continuous repair process preserves the functional integrity and strength of the adult skeleton through cycles of bone remodeling. Four cell types are involved in bone modeling and remodeling: chondrocytes, osteoblasts, osteocytes, and osteoclasts. Chondrocytes are the initial cell type that emerges during development. In early embryogenesis, mesenchymal precursors create a mold and differentiate into chondrocytes, which proliferate and excrete a matrix containing elastin and type II collagen to form a cartilaginous analog of the bone [6,7].

Osteoblasts constitute 4–6% of bone cells. They originate from multipotent mesenchymal stem cells, which have the potential to differentiate into chondrocytes, osteoblasts, or adipocytes. Osteoblasts typically have a lifespan of about two weeks, except for those that become bone-lining cells or osteocytes. Under normal physiological conditions, osteoblasts produce type I collagen fibers, which make up 90% of the organic matrix of bone. Additionally, osteoblasts secrete proteins that regulate the assembly of collagen fibers and facilitate mineralization [8]. Osteocytes comprise 90% to 95% of bone cells and originate from osteoblasts embedded within the bone matrix. As mechanosensors, osteocytes regulate bone modeling and remodeling by controlling osteoclasts through the RANKL/RANK pathway and influencing osteoblasts via Wnt signaling modulation. Additionally, osteocytes are critical in regulating phosphate homeostasis [9].

Osteoclasts comprise only 1% to 2% of bone cells and are responsible for resorbing bone matrix and minerals. They attach to the bone surface, requiring resorption, and form a sealing zone where the osteoclast polarizes into a ruffled border and a basolateral membrane. Carbonic anhydrase II (a specific osteoclast pump) and chloride channels create an acidic environment within the resorption lacunae, dissolving hydroxyapatite to release calcium and phosphate ions. Simultaneously, secreted cysteine proteases (such as cathepsin K) digest the organic components of the bone [10].

The bone remodeling cycle, influenced by the hormone parathyroid hormone (PTH), consists of a catabolic phase (bone resorption) and an anabolic phase (new bone formation). This ongoing repair process maintains the functional integrity and strength of the adult skeleton through the bone remodeling cycle. The basic multicellular unit involved in bone remodeling comprises osteoclasts and osteoblasts, whose activities are regulated by osteocytes. This dynamic interaction ensures bone tissue’s continuous renewal and maintenance, which is critical for skeletal health and function [11].

Macrophages also play a crucial role in osteogenesis, participating in various stages, from cellular damage to the inflammatory and osteogenic phases. M2 macrophages are primarily responsible for promoting osteoblast formation, while M1 macrophages are essential for recruiting mesenchymal stem cells (MSCs) that differentiate into osteoblasts. M1 macrophages release pro-inflammatory cytokines that enhance osteoclast activity and recruit MSCs, while M2 macrophages secrete anti-inflammatory cytokines that upregulate RUNX-2 in MSCs, facilitating their differentiation into osteoblasts [12]. Furthermore, a study demonstrated that silencing or overexpressing LMW-PTP significantly affected osteoblast adhesion. Specifically, silencing LMW-PTP led to increased phosphorylation of FAK and Src. At the same time, overexpression of the osteoblast decreased phosphorylation levels, indicating that LMW-PTP plays a vital role in modulating these signaling pathways during osteoblast cell adhesion [13].

Under basal conditions, osteocytes secrete TGF-β and sclerostin, inhibiting osteoclastogenesis and bone formation by Wnt-activated osteoblasts [14]. Increased mechanical loading or local micro-damage decreases local TGF-β levels and activates bone cells to recruit osteoclast progenitors [15]. Osteocytes and bone-lining cells express macrophage colony-stimulating factor (M-CSF) and RANKL, both essential for osteoclastogenesis [16]. RANKL induces changes in osteoclast migration, survival, attachment to the bone surface, and sealing zone formation. Additionally, osteoblasts and bone marrow stromal cells express osteoprotegerin (OPG), which inhibits the RANK-RANKL signaling pathway [17]. The ratio of RANKL to OPG is crucial in determining the differentiation and activity of osteoclasts. Local cytokines and systemic hormones that regulate bone remodeling include TNF-α, IL-1, prostaglandin E2, estrogen, parathyroid hormone (PTH), and glucocorticoids. The resorption phase lasts for 30–40 days. Paracrine signals released from the degraded matrix recruit osteoblasts, who initiate bone formation over the subsequent 150 days. Osteoblasts secrete and mineralize new bone matrix (osteoid) to fill the resorption cavities. During bone formation, some osteoblasts become embedded in the newly formed bone and undergo terminal differentiation into osteocytes. These osteocytes’ secretion of sclerostin and other inhibitors halts bone formation, returning to a quiescent state where osteoblasts become bone-lining cells [6,7].

## 3. Interaction of Bone Mineral Density, CTX-1, and Osteocalcin

### 3.1. Bone Mineral Density

BMD (bone mineral density) testing is crucial for diagnosing osteoporosis, assessing future fracture risk, and monitoring treatment effectiveness [18]. Trabecular bone exhibits a higher metabolic rate than cortical bone, making regions with a high trabecular-to-cortical ratio the ideal sites for BMD measurements. These areas, known as regions of interest, typically include the axial skeleton, which is preferred for screening due to significant changes in trabecular density. However, other sites like the forearm, tibia, and calcaneus are also suitable for BMD analysis [19].

DEXA (Dual-Energy X-ray Absorptiometry) is the preferred method for assessing BMD due to its efficiency, which is characterized by high precision, quick execution, and minimal radiation exposure. It is commonly used to measure BMD at key sites like the lumbar spine and femur. It provides absolute BMD measurements (g/cm^2^), essential for consistent tracking and comparison across studies [20]. Despite these advantages, DEXA’s primary limitation is the potential for measurement inconsistencies, with variability that can reach up to 20% between scans [19].

A BMD T-score of ≤−2.5 at the hip defines osteoporosis, while a T-score between −1 and −2.5 defines osteopenia. Additionally, patients with fragility fractures are classified as having osteoporosis regardless of their T-score [21]. Factors affecting DEXA BMD results include significant weight changes, absolute body size, and measurement variances specific to the DEXA machine used. The variability inherent in DEXA machines means that significant changes in BMD must exceed 0.055 and 0.045 g/cm^2^ at the spine and hip, respectively, corresponding to about a 4–7% change in serial BMD measurements, depending on the initial BMD values [18].

### 3.2. The C-Terminal Telopeptide of Type I Collagen

The C-terminal telopeptide of type I collagen (CTX-1) is a degradation product of Type 1 collagen generated by the enzymatic activity of cathepsin K. CTX exists in the α and β isomerized forms. These isomerized forms undergo further isomerization to form the D and L forms. Spontaneous β isomerization of the α isoform occurs with protein aging. Therefore, changes in the ratio of the α and β isomer forms occur with new bone formation in physiological conditions such as childhood growth and pathological conditions such as malignant bone diseases [22].

The International Osteoporosis Foundation and the International Federation of Clinical Chemistry and Laboratory Medicine have proposed serum CTX-1 as a reference marker for bone resorption to assess fracture risk and monitor therapy [4]. Serum CTX-1 levels (a marker of osteoclastic activity) have been shown to inversely correlate with the efficacy of drugs in suppressing bone resorption. CTX-1 levels in patients with fragility fractures were higher in patients with persistent treatment, supporting the use of CTX-1 for monitoring bone resorption suppression [23]. Another study by Bjerre-Bastos et al. successfully demonstrated the usefulness of CTX-1 and CTX-2 in predicting the need for total joint replacement in cases of osteoarthritis [24]. The main issue with measuring CTX is its circadian variation, with a peak in the early morning (around 5:00 AM) and a nadir in the afternoon (around 2:00 PM). Additionally, food intake affects CTX levels, with postprandial levels being 20% lower than fasting. Therefore, CTX-1 sampling is recommended in the morning after an overnight fast [4].

### 3.3. Osteocalcin

Osteocalcin (OCN) is a hydroxyapatite-binding protein synthesized exclusively by osteoblasts, odontoblasts, and hypertrophic chondrocytes, serving as a marker of osteoblastic activity and bone formation. OCN constitutes 15% of the non-collagenous bone matrix and indicates bone formation. OCN undergoes post-translational modifications, one of which involves the carboxylation of specific amino acid residues. The carboxylated form of OCN is known as carboxylated OCN (OCN) and is found in bone. In contrast, the uncarboxylated form is undercarboxylated OCN (ucOCN or n-mid osteocalcin) and is present in circulation. N-mid osteocalcin refers explicitly to the N-terminal or amino-terminal portion of the OCN molecule. This region is prone to cleavage during the carboxylation process, and the resulting fragments, including N-mid osteocalcin, can be used as markers to assess bone turnover [25,26].

Using OCN as a biomarker for bone formation offers advantages such as tissue specificity, wide availability, and low variability. Serum OCN, as a bone remodeling biomarker, can help assess osteoporosis and predict fracture risk in elderly individuals, particularly in women [27]. However, the utility of OCN is limited by its short half-life, instability of the intact molecule, and influence on vitamin K status, renal function, and the circadian rhythm [28]. Additionally, samples for OCN measurement have specific collection and transportation requirements due to OCN’s instability. It is recommended that samples be stored at approximately four °C and processed within 4 h of collection. Consistent hemolysis of samples can reduce OCN levels, likely by increasing OCN degradation. Due to this instability, caused mainly by the labile C-terminal sequence of six amino acids, the measurement of the N-Mid-OCN fragment (amino acids 1–43) has shown promising clinical utility [25,29].

### 3.4. Interplay of BMD, CTX-1, and OCN

**Figure 1:**
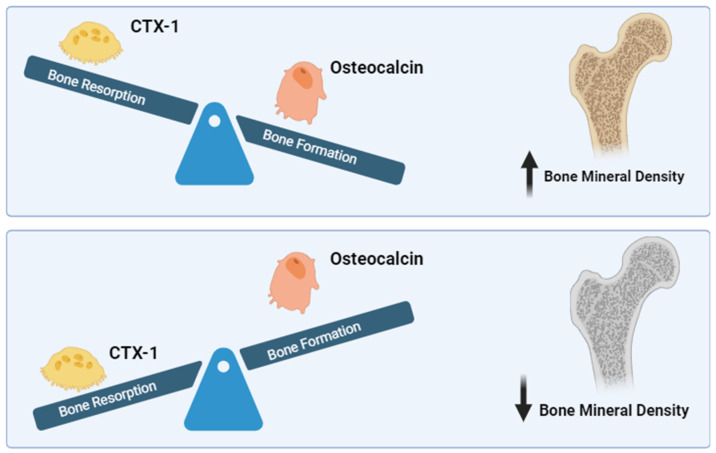
Interaction of CTX-1, osteocalcin, and bone mineral density. Osteoclasts produce CTX-1, which is a marker of bone resorption. Osteoblasts produce osteocalcin, which is a marker of bone formation. When the bone formation rate surpasses the bone resorption rate, bone mineral density (BMD) increases. On the other hand, when the bone resorption rate surpasses the bone formation rate, BMD decreases. CTX-1: C-terminal telopeptide of type I collagen. Created in BioRender.

The relationship between BMD, CTX-1, and OCN involves complex interactions within the bone remodeling process (Figure 1). Osteoblasts and osteoclasts are key cells in bone remodeling, with osteoblasts responsible for bone formation and osteoclasts for bone resorption. Bone mineral density (BMD) is a crucial measure that reflects the balance between bone formation and resorption, which is primarily regulated by osteoblasts and osteoclasts. BMD increases when bone formation by osteoblasts surpasses bone resorption by osteoclasts, leading to stronger bones. Conversely, when resorption outpaces formation, BMD decreases, indicating bone loss [30].

Furthermore, CTX-1 is a well-established marker of bone resorption. It is released into the bloodstream during bone remodeling when osteoclasts break down the C-terminal region of type I collagen, the most abundant protein in bone. CTX-1 levels in the serum are indicative of osteoclastic activity and can be used to monitor the effectiveness of treatments. The specificity of CTX-1 as a marker comes from its release during the cathepsin K-mediated degradation of bone collagen, distinguishing it from other resorption markers that may be influenced by different metabolic pathways [27,29,31].

On the other hand, OCN is a protein produced by osteoblasts during bone formation and is widely recognized as a marker of bone turnover. Specifically, the undercarboxylated form of osteocalcin, known as N-mid OCN, is released into the bloodstream and plays a crucial role in reflecting the rate of bone turnover. This marker is significant because it also interacts with various signaling pathways, including those related to energy metabolism and insulin sensitivity [32]. The endocrine functions of OCN have been linked to metabolic processes such as glucose regulation and fat metabolism, highlighting its broader role beyond bone health [33,34,35]. The measurement of N-mid OCN in clinical settings can provide valuable information on bone health and metabolic functions, making it a useful biomarker in assessing conditions like osteoporosis metabolic syndromes.

## 4. Role of Tyrosine Kinase in Bone Metabolism

All types of malignancies can cause disruptions in kinase signaling. Tyrosine-protein kinases catalyze the phosphorylation of specific tyrosine residues and are critical regulators of signaling pathways that involve cell proliferation, differentiation, and apoptosis. These proteins exhibit constitutive tyrosine kinase activity and stimulate hematopoietic transformation and myeloproliferative activity. The dominant isoform, BCR-Abl, is a protein found in over 90% of patients with chronic myelogenous leukemia (CML). This protein’s unregulated kinase activity is central to the pathogenesis of CML, driving the aberrant growth and survival of leukemic cells [36]. BCR-Abl is identified in the Philadelphia chromosome, a hallmark of chronic myelogenous leukemia (CML). The chromosomal translocation between chromosomes 9 and 22 forms the BCR-Abl tyrosine kinase, which constitutively activates Abl kinase through the BCR promoter. This leads to continuous proliferative signaling. This aberrant kinase activity disrupts standard cellular control mechanisms, driving the unchecked proliferation of myeloid cells characteristic of CML [37].

Recent research has shown that tyrosine kinases play an important and vital role in the regulation of bone metabolism; specifically, osteoblasts and osteoclasts that are responsible for bone formation as well as bone and resorption, respectively. Among these kinase families, the Src family is particularly prominent. The function of osteoclasts depends on the activity of Src kinase, which also inhibits bone synthesis by osteoblasts. Osteoporosis develops in mice that lack Src due to impaired function of osteoclasts and increased bone formation, indicating the dual effect of Src on resorption promotion and formation inhibition [38]. Another crucial one is MerTK kinase, which was found to negatively regulate β-catenin and Smad signaling pathways during the early stages of bone development while suppressing osteoclastogenesis through an elevated OPG/RANKL ratio. This results in an increase in bone volume but a reduction in bone resorption in Mertk knockouts, hence suggesting therapeutic potential for targeting MerTK for the treatment of such lytic conditions as osteoporosis [39]. Moreover, non-receptor tyrosine kinase Matk, which inhibits Src, modulates osteoclast and osteoblast activities independently and through c-Src [40]. Therefore, tyrosine kinases can be considered bone regulators or controllers of bone mass.

## 5. Impact of Tyrosine Kinase Inhibitors to Bone Turnover

Since tyrosine kinase plays a significant role in bone remodeling of bone mass, tyrosine kinase inhibitors (TKIs) consequently affect the bone remodeling process. Imatinib is found to inhibit MSC proliferation from which osteoblasts originated [41]. Dasatinib appears to direct differentiation away from the adipocyte lineage and toward the osteoblast lineage [42]. VEGF inhibitors (Sunitinib and Cabozantinib) and RET inhibitors (Vandetanib) have been shown to interfere with the osteoblast differentiation process [43,44]. Fully differentiated osteoblasts are known as osteocytes and, along with immature osteoblasts, secrete RANKL, the primary driver of osteoclast differentiation and activity [45]. However, the effects of TKIs on osteocytes are largely unknown [46].

TKIs can cause an increase in parathyroid hormone (PTH) levels. Several studies have reported that TKIs, particularly those like Imatinib, Dasatinib, and Sunitinib, may lead to secondary hyperparathyroidism [46]. This condition occurs because TKIs can reduce calcium levels by inhibiting osteoclast activity, which decreases bone resorption and subsequently lowers serum calcium. The body responds to this drop in calcium by increasing the secretion of PTH to maintain calcium homeostasis [47]. A tonic increase in PTH leads to increased RANKL production, which is expected to increase osteoclast differentiation and activity [48]. However, despite hyperparathyroidism, some TKIs have been found to inhibit osteoclast activity [37]. Osteoclasts originate from hematopoietic stem cells in the bone marrow and are stimulated by factors including RANKL to differentiate into mature multinucleated cells. Src kinase has been shown to enhance osteoclast differentiation and activity; Dasatinib is a potent Src kinase inhibitor [49]. M-CSF-induced pre-osteoclast differentiation into mature osteoclasts is inhibited by Sunitinib and Imatinib [50,51].

## 6. Impact of Imatinib to BMD, CTX-1, and Osteocalcin

Recent studies have indicated changes in bone and mineral metabolism in patients receiving Imatinib, with secondary hyperparathyroidism and decreased bone remodeling observed during long-term Imatinib treatment. In vitro studies have revealed that Imatinib enhances bone formation in CML patients [36,52,53]. A longitudinal study on 17 CML patients found that serum parathyroid hormone levels increased over four years, with 7 out of 17 patients developing secondary hyperparathyroidism. However, mean areal and volumetric BMD remained stable and even higher in the cortical compartment than controls [54].

Imatinib is thought to affect bone mineral metabolism through the inhibition of other tyrosine kinases present in bone cells, including PDGFRa and PDGFRb, C-kit, and the c-fms receptor on monocytes and macrophages [37]. Dib et al. suggested that Imatinib may influence mature osteoclasts through c-fms inhibition, and Imatinib may have clinical value in treating diseases where bone destruction occurs due to excessive M-CSF production, such as osteoporosis, inflammation-induced osteolysis, and tumor-induced osteolysis [51]. A study by Dewar et al. in mice also found that Imatinib might be an effective antiosteolytic agent. Imatinib inhibits osteoclast formation and activity in vivo. The inhibition of osteoclast differentiation is likely due to the inhibition of c-fms signaling, while the inhibition of osteoclast function occurs indirectly through decreased RANK expression [55].

On the other hand, studies in mice have shown a narrowing of the growth plate in the proximal tibia of animals treated with Imatinib. Imatinib has antiresorptive effects on osteoclasts that interfere with the length of tubular bones, particularly in prepubertal animals [36]. A study by O’Sullivan found that Imatinib does not affect BMD in the long term. This aligns with recent studies in healthy mice where Imatinib administration did not increase BMD or alter biochemical markers of bone resorption. Although Imatinib promotes early osteoblast differentiation, it also reduces mineralization, which is more pronounced at low Imatinib concentrations. The effect of Imatinib on osteoblast differentiation also depends on the cell’s maturation stage. Therefore, it appears that CML patients initially experience increased bone formation with Imatinib therapy, which does not persist after the first 1 or 2 years of treatment [56,57]. This was evidenced in a study by Choeyprasert et al. on six CML children, which found a correlation between low BMD without affecting bone parameters and a high prevalence of vitamin D deficiency [58].

Several studies have found a direct correlation between Imatinib use and the bone turnover markers CTX-1 and N-mid OCN. Jaeger et al. found that in 17 children with CML undergoing prolonged Imatinib therapy, serum CTX-I levels were above the normal range in 57% of patients, and N-mid OCN levels showed a significant linear decrease of −0.30 µg per week (*p* = 0.04) [59]. Tauer also observed a significant decrease in N-mid OCN in mice treated with Imatinib for ten weeks, although no difference was found in CTX-1 levels [60]. We summarize the results of the studies in Table 1.

## 7. Rationale for Combination of BMD, Osteocalcin, and CTX-1 to Monitor Bone Remodeling

BMD examination with DEXA is the gold standard for diagnosing osteoporosis and monitoring treatment. However, changes in BMD are slow and require at least 1 year to assess treatment efficacy. Although BMD measured by DEXA is used to diagnose osteoporosis according to WHO recommendations, bone quality also determines bone strength and fracture risk. Furthermore, some clinical trials have shown that the reduction in fracture risk associated with anti-resorptive therapy can occur independently of changes in BMD. Therefore, for a complete assessment of bone strength, BMD should be combined with an evaluation of bone quality. One important contributor to bone strength is the rate of bone remodeling, which can be assessed by measuring bone turnover markers (BTMs) [61].

Bone turnover markers (BTMs) are a series of proteins or protein-derived biomarkers released during bone remodeling by osteoblasts or osteoclasts. BTMs can provide prognostic information regarding fracture risk that complements radiographic measurements of bone mass. However, testing using BTMs must consider a large number of preanalytical factors and comorbid clinical conditions that affect BTM levels. BTMs respond quickly to changes in bone physiology, making them useful for determining patient response and adherence to osteoporosis therapy [29].

Osteoclasts secrete TRAP and Cathepsin K, which metabolize collagen I into DPD, PYR, NTX, and CTX metabolites. Meanwhile, osteoblasts produce BAP, pro-collagen, and OCN. Therefore, both can be used as markers of bone formation and resorption or bone turnover markers. Wu et al. stated that in relation to the low adherence rates to osteoporosis treatment, experts reached a consensus on the use of BTMs to increase awareness and short-term monitoring of osteoporosis treatment in the Asia-Pacific region [61]. The experts support the use of BTMs, particularly CTX-1 and PINP, as short-term monitoring tools to help physicians assess responses to osteoporosis therapy and make early adjustments to treatment regimens before BMD examinations. The absolute value or rate of change from baseline BTMs can be used to monitor the efficacy of osteoporosis therapy [61]. A systematic review by Hong et al. also found that changes in OCN are useful in evaluating long-term changes in BMD after anti-osteoporosis drug interventions [62].

## 8. Conclusions

Imatinib and other TKIs change the activity of both osteoclast and osteoblast cells, and therefore, the risk for bone side effects should be managed properly. In the case of CML patients on Imatinib, the assessment of bone health, a combination of BMD and bone turnover marker assays such as CTX-1 and osteocalcin, can be used to help in determining bone-modeled changes earlier than BMD.

## Figures and Tables

**Table 1:** Summary of study about impact of Imatinib on BMD, CTX-1, and osteocalcin.

Study/Authors	Study Type	Participants/Subjects	Key Observations
Jönsson et al. [54]	Longitudinal Study	17 CML patients	A total of 7/17 developed secondary hyperparathyroidism; increased serum parathyroid hormone levels over four years; mean areal and volumetric BMD remained stable; cortical BMD higher than controls.
Dib et al. [51]	Clinical/Preclinical	Not specified	Imatinib influences mature osteoclasts through c-fms inhibition; potential clinical value in treating osteoporosis and osteolysis.
Dewar et al. [55]	Animal Study	Mice	Imatinib inhibits osteoclast formation and activity in vivo. It is an effective antiosteolytic agent and affects c-fms signaling and RANK expression.
O’Sullivan [56]	Animal Study	Healthy mice	Early osteoblast differentiation promoted reduced mineralization at low concentrations. There is no long-term effect on BMD, and it does not alter bone resorption markers.
Choeyprasert et al. [58]	Clinical Study	44 CML children	Correlation with low BMD without affecting bone parameters; high prevalence of vitamin D deficiency; Imatinib linked to low BMD and vitamin D deficiency.
Jaeger et al. [59]	Clinical Study	17 children with CML on prolonged Imatinib	Decrease in N-mid OCN; CTX-I levels high in a majority of patients.
Tauer et al. [60]	Animal Study	Mice	Similar findings as Jaeger et al. in terms of N-mid OCN.

## Data Availability

All data are available upon request.
